# Current strategies for quantification of estrogens in clinical research

**DOI:** 10.1016/j.jsbmb.2019.04.022

**Published:** 2019-09

**Authors:** Nina Denver, Shazia Khan, Natalie Z.M. Homer, Margaret R. MacLean, Ruth Andrew

**Affiliations:** a Mass Spectrometry Core, Edinburgh Clinical Research Facility, Queen’s Medical Research Institute, 47 Little France Crescent, Edinburgh, EH16 4TJ, United Kingdom; b Institute of Cardiovascular and Medical Sciences, College of Medical, Veterinary and Life Sciences, University of Glasgow, University Avenue, Glasgow, G12 8QQ, United Kingdom; c Strathclyde Institute of Pharmacy and Biomedical Sciences, University of Strathclyde, 161 Cathedral Street, Glasgow, G4 0RE, United Kingdom; d University/BHF Centre for Cardiovascular Science, Queen's Medical Research Institute, University of Edinburgh, 47, Little France Crescent, Edinburgh, UK, EH16 4TJ

**Keywords:** 17βHSD1 and 17βHSD2, 17beta-hydroxysteroid dehydrogenase type 1 & 2, PPZ, 1-(2,4-dinitro-5-fluorophenyl)-4-methylpiperazine, MPPZ, 1-(2, 4-dinitrophenyl)-4,4-di- methylpiperazinium, MIS, methylimidazole-2-sulfonyl chloride, DMIS, 1,2-dimethylimidazole-5-sulfonyl chloride, FMP, 2-fluoro-1-methyl-pyridinium *p*-toluene sulfonate, 2,4 or 16-OHE2, 2, 4 or 16-hydroxestradiol, 2,4 or 16-OHE1, 2, 4 or 16-hydroxestrone, DNBF, 2,4-dinitrofluorobenzene, 2 or 4-MeOE2, 2 or 4-methoxyestradiol, 2 or 4-MeOE1, 2 or 4-methoxyestrone, BMP, 3-bromomethyl-propyphenazone, APZ, 4-(4-methyl-1-piperazyl)-3-nitrobenzoyl azide, NBCOCL, 4-nitrobenzoyl chloride, 2OHE-3ME, 2-hydroxyestrone-3-methyl ether, 16epiOHE2, 16β-hydroxy-17β-estradiol, 16ketoOHE2, 16-oxo-17β-estradiol, 17epiOHE2, 16α-hydroxy-17α-estradiol, APCI, atmospheric pressure chemical ionization, APPI, atmospheric pressure photoionization, COMT, catechol-*O*-methyltransferase, CI, chemical ionization, CYP, cytochrome P450, DS, dansyl chloride, DT-IMS, drift tube-ion mobility mass spectrometry, E2, estradiol, E1, estrone, EOC, ethoxycarbonlyation, FA/D-IMS, field asymmetric/differential- ion mobility mass spectrometry, GC–MS/MS, gas chromatography tandem mass spectrometry, HFB, heptafluorobutyryl chloride, OHE, hydroxyestrogens, IMS, ion mobility mass spectrometry, IS, internal standard, LC–MS/MS, liquid chromatography tandem mass spectrometry, LLE, Liquid Liquid Extraction, C1-NA-NHS, *N*-methyl-nicotinic acid *N*-hydroxysuccinimide ester, TMSI, *N*-(trimethylsilyl)imidazole, NMPS, *N*-methyl pyridinium-3-sulfonyl chloride, MSTFA, *N*-methyl-*N*-(trimethylsilyl)-trifluoroacetamide, PED, *N*’-(5-fluoro-2,4-dinitrophenyl)-*N*,*N*-dimethyl-1,2- ethanediamine, NS, Not stated, PDFO, pentadecafluorooctanoyl chloride, PFBO, perfluorobenzoyl chloride, PFBHA, pentaflurobenzoyl hydroxylamine hydrochloride, P, picolinoyl carboxylate, PS, pyridine-3-sulfonyl chloride, SPE, solid phase extraction, TQ-S, tandem quadrupole mass spectrometry, TW-IMS, travelling wave-ion mobility mass spectrometry, TFA, trifluoracetic acid, UFLC, ultraflow LC, Estrogen, Liquid chromatography tandem mass spectrometry, Gas chromatography tandem mass spectrometry, Extraction, Derivatization

## Abstract

Estrogens and their bioactive metabolites play key roles in regulating diverse processes in health and disease. In particular, estrogens and estrogenic metabolites have shown both protective and non-protective effects on disease pathobiology, implicating the importance of this steroid pathway in disease diagnostics and monitoring. All estrogens circulate in a wide range of concentrations, which in some patient cohorts can be extremely low. However, elevated levels of estradiol are reported in disease. For example, in pulmonary arterial hypertension (PAH) elevated levels have been reported in men and postmenopausal women. Conventional immunoassay techniques have come under scrutiny, with their selectivity, accuracy and precision coming into question. Analytical methodologies such as gas and liquid chromatography coupled to single and tandem mass spectrometric approaches (GC–MS, GC–MS/MS, LC–MS and LC–MS/MS) have been developed to quantify endogenous estrogens and in some cases their bioactive metabolites in biological fluids such as urine, serum, plasma and saliva. Liquid-liquid or solid-phase extraction approaches are favoured with derivatization remaining a necessity for detection in lower volumes of sample. The limits of quantitation of individual assays vary but are commonly in the range of 0.5–5 pg/mL for estrone and estradiol, with limits for their bioactive metabolites being higher. This review provides an overview of current approaches for measurement of unconjugated estrogens in biological matrices by MS, highlighting the advances in this field and the challenges remaining for routine use in the clinical and research environment.

## Highlights

• Profiling estrogens and their metabolites by mass spectrometry (MS) offers insights into health and disease.
• Low limits of quantification can be achieved by MS approaches, interfaced with GC or LC.
• Improvements in recovery, ion suppression and detection are discussed.
• Advances in current technologies for future method development strategies are proposed.

## 1 Introduction

### 1.1 Estrogen biochemistry

**Fig. 1:**
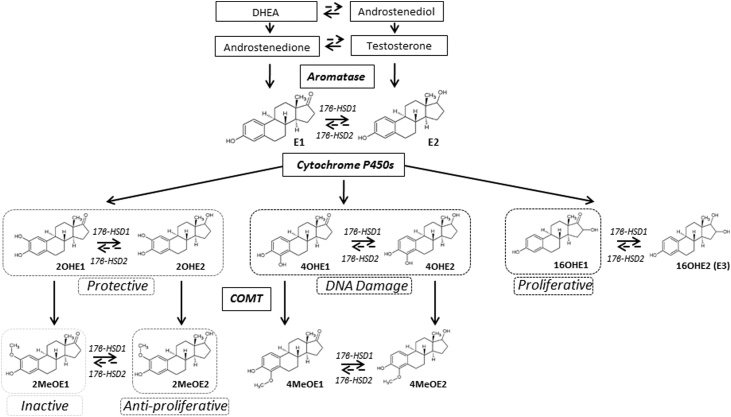
Endogenous steroid hormone pathway; formation of endogenous estrogen from sex hormone substrates dehydroepiandrosterone (DHEA), andostenediol (A5), androstenedione (A4) and testosterone (T). Oxidative metabolism of estrone (E1) and estradiol (E2) at C2, C4 and C16 positions by cytochrome P450 enzymes leads to the generation of hydroxyestrogen metabolites (2OHE, 4OHE & 16OHE). The 2OHE and 4OHE metabolites are rapidly converted to the methoxyestrogens by catechol-O-methyltransferases (COMT). E1 and E2 metabolites are maintained in equilibrium through the actions of 17β-hydroxysteroid dehydrogenase 1 & 2 enzymes. Dysregulation in the balance of these metabolic pathways can be both protective and non-protective, examples being in pathophysiology of pulmonary arterial hypertension and cancer.

Estrone (E1) and estradiol (E2) are the predominant circulating female sex steroids with multiple functions throughout the body. The third most common form in humans, estriol (E3 or 16OHE2), can be produced from estradiol or from estrone, the latter via the 16-hydroxyestrone (16OHE1) intermediate [1]. Estrogens can be synthesized on demand in some tissues from the major circulating adrenal steroids dehydroepiandrosterone (DHEA), andostenediol (A5), through androstenedione (A4) and testosterone (T) [2] via the enzyme aromatase, Fig. 1. Ovaries are the main production site of estrogens in premenopausal women, whereas tissues such as adipose play a greater role post-menopausally [3]. Estradiol is the most abundant hormone in pre-menopausal women, whereas estrone is more prevalent in post-menopausal women and also in males, being generated from adrenal androstenedione. Isomers of estradiol exist in α and β configurations; 17β-estradiol (E2) refers to the main bioactive version whilst 17α-estradiol is generally thought to be less active [4]. Estriol increases throughout pregnancy being generated in the placenta. Aromatic oxidation of estrone and estradiol generates hydroxy-metabolites which are converted to methoxylated metabolites, but the exact circulating level of each remains largely unknown and under-investigated [5]. The levels of the bioactive metabolites are assumed lower than the main circulating estrogens, Table 1.

**Table 1:** Reference ranges of concentrations of estrogens in human plasma.

Group	Age	Estrone	Estradiol	Estriol
	(Y)	(pg/mL)	(pg/mL)	(pg/mL)
Female Children	0-15	ND - 200	ND - 40	ND
Premenopausal	18-55	17 - 200	15 - 350	<80
Pregnancy	–	>187	188 - 7192	>2100
Postmenopausal	>55	7 - 40	<10	ND
Male Children	0-18	ND - 46	ND - 38	ND
Males	>18	10 - 60	10 - 40	<70

Concentration guidelines from Mayo medical laboratories (https://www.mayocliniclabs.com/test-catalog/Clinical+and+Interpretive/84230 and www.mayocliniclabs.com/test-catalog/Clinical+and+Interpretive/81711 accessed 15/07/2019); Children <18 Y; ND = Not detected; Y = years.

Throughout this review all estrogens and metabolites will be referred to collectively as estrogens. Estrone and estradiol are in equilibrium by interconversion by 17βHSD1 and 17βHSD2 enzymes, catalyzing reduction or oxidation respectively at the C17 position ketone/hydroxyl, and the balance largely favours estrone formation, Fig. 1. Further metabolism occurs via cytochrome P450 (CYP) enzymes, generating bioactive hydroxy metabolites upon oxidation of the parent molecules at the C16, C4 and C2 positions listed in the order of reactive preference [6]. In the C16 position, oxidation can occur via CYP1B1 [7] and 16OHE1 formed can be interconverted with 16OHE2. Both 16-hydroxy estrogens are further metabolised by conjugation. The 2- and 4-hydroxy-estrone and estradiol metabolites, collectively known as catechol estrogens (2OHE1, 4OHE1, 2OHE2 & 4OHE2), are rapidly converted (t 1/2 = 90 min [8]) to 2- and 4-methoxy–estrone and estradiol metabolites (2MeOE1, 4MeOE1, 2MeOE2 and 4MeOE2) by the action of catechol-*O*-methyltransferase (COMT). Hydroxy and methoxy-estrone and estradiol metabolites are also maintained in constant equilibrium by 17βHSD1 and 17βHSD2 enzymes prior to their respective metabolism and removal from the body mainly in the liver. Estrogens are converted to glucoronide and sulfate conjugates and catechol metabolites also form glutathione conjugates, all potential mechanisms of detoxification in hepatic and extra-hepatic sites [9].

### 1.2 Influence of estrogens in disease pathobiology

Epidemiological and experimental studies implicate estrogens in a number of diseases with the potential roles of bioactive metabolites becoming more prominent. For example, in cancer and cardiovascular fields estradiol, 16OHE1, 2OHE2 and 4OHE2 have been implicated in disease progression and 2OHE1, 4OHE1, 4MeOE1, 2MeOE2 and 4MeOE2 have shown protective roles. The remaining 2MeOE1 is thought to be inactive [5,10]. At site specific locations prior to removal, estrogens and the bioactive metabolites may act through genomic signalling cascades via estrogen receptors (ER) or alternatively by rapid non-genomic actions via G-protein coupled estrogen receptor 1 (GPER) and may directly alter protein signalling [11].

Elevated estrogen levels in serum and plasma of women in particular have been associated with increased risk of breast [12,13], endometrial [14,15] and ovarian cancers [16,17], whilst in males estrogen-androgen imbalance is thought significant in the development of aggressive prostate cancers [18]. Emerging evidence implicates estrogen metabolism in the aetiology of diabetes [19], possibly explaining why in breast cancer obesity has proven to be a major contributing risk factor [20]. Most literature addresses estradiol and estrone; the bioactive estrogen metabolites remain less studied but are now gaining more prominence in each field. For example higher 16-hydroxy estrogen production has been linked to greater risk of diseases such as prostate cancer and pulmonary arterial hypertension (PAH) [18].

In oncology, several factors link predisposition to metastasis to estrogen bioactivity. Clinically, three main scenarios are presented; in late menopause when site specific estrogen production and metabolism becomes more prominent; in hormone replacement therapy use where metabolic dysfunction occurs via increased exogenous supply; and thirdly in the presence of specific single nucleotide polymorphisms (SNPs) in aromatase that result in increased circulating plasma estradiol levels [12]. All scenarios lead to exacerbation of breast cancer symptoms [10]. Over and above changes in estrone and estradiol signalling, changes in urinary estrogen metabolite levels have been reported in mammary tumours with an emphasis on the 2/16-hydroxyestrogen ratio [21]. Here, higher levels of 2-hydroxyestrogens compared to their mitogenic counterparts, 16-hydroxyestrogens, are associated with decreased risk of tumour growth and disease progression [22]. Specific bioactive metabolites are also linked to cardiovascular disease [[23], [24], [25]] with striking similarities to oncology findings. In PAH, a disease underpinned by gender differences, understanding the actions of estrogens and their metabolites may be key in elucidating the cause for female predominance. Research, *in vitro* and *in vivo* has linked 16OHE1 to cellular proliferation and vascular remodelling, significant phenotypic hallmarks of PAH [[26], [27], [28]]. 16OHE1 exhibits a higher binding affinity and estrogenic potency than parent molecules at classical estrogen receptors [29], potentially activating the classical genomic signalling cascade and playing a pathogenic role in the pulmonary circulation. In this setting, increased levels in urine [30] also coincide with induction of smooth muscle proliferation within the pulmonary arteries [27]. Alternatively, metabolites like 2MeOE2 have demonstrated protective effects via disruption of HIF1α signalling, decreasing mitogenic proliferative effects within lung fibroblasts in an apoptotic manner [31]. Interestingly a recent comprehensive study has further implicated elevated estradiol levels in male patients with PAH, linking this to poorer clinical outcomes [32]. Therefore, the potential consequences of estrogenic hormone imbalance within the body, at site-specific locations in both females and males, prompt investigation of the diverse profile of circulating estrogens with the aim of developing targeted therapeutic modulators within this sex hormone pathway.

## 2 Quantification of estrogens

This review will discuss approaches for analysis of unconjugated estrogens.

### 2.1 Immunoassays

Measurement of circulating estrogens in clinical diagnosis, research and monitoring often involves enzyme linked immunoassays (ELISAs) and radioimmunoassays (RIAs) [33,34] and has largely focussed on estradiol and estrone. Mainly these techniques are chosen due to their low cost and routine nature [35]. Both rely on the action of an antigen (estrogen) binding to specific antibodies; for ELISA, the detection of this interaction is accomplished via incubation with a substrate(s) known to emit a measurable product; for RIA, radioactive scintillation counting is applied. These methods can lack selectivity, being dependent on antibody characteristics, often exhibiting cross reactivity between different estrogens of interest and other species. This problem is particularly marked when measuring lower levels. High selectivity at low concentrations is a critical requirement for accurate analysis of estrone and estradiol particularly in men and older women, and the same rigour is needed to assay low levels of bioactive estrogen metabolites [36,37]. Several studies for more abundant steroidal compounds such as cortisol, testosterone and vitamin D illustrate an imprecision between reported concentrations and a bias for false positives using immunoassays over a number of analytical methods [[38], [39], [40]]; this has led to the Endocrine Society issuing a consensus statement recommending avoidance of immunoassays for steroid hormone assays [41]. Therefore, development of robust analytical techniques capable of simultaneous quantification of panels of estrogens at low circulating concentrations becomes justified and here hyphenated mass spectrometry techniques have led the way.

### 2.2 Analytical approach

**Fig. 2:**
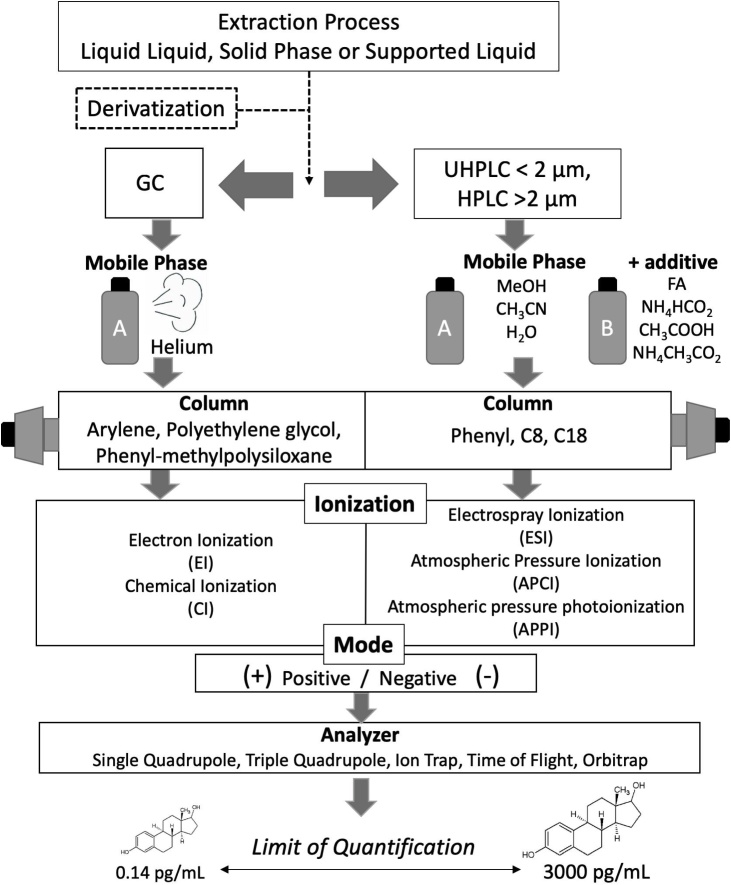
Schematic workflow for analysis of estrogens by mass spectrometry.

The journey of estrogen quantification began with researchers exploring a wide variety of diverse matrices using high performance liquid chromatography (HPLC), with a few publications reporting quantification of pharmaceutical estrogens within bio-fluids [42]. Transfer of methods for analysis of endogenous steroids by HPLC has proven difficult, due to insufficient sensitivity. The majority of analytical technologies that became available for clinical analysis of estrogens originally employed gas chromatography mass spectrometry (GC–MS). In more recent years, with the evolution of narrower bore liquid chromatography columns with smaller particle sizes, liquid chromatography tandem mass spectrometry (LC–MS/MS) has increasingly featured. Both approaches benefit from the use of stable isotope internal standards (IS) and have levels of specificity unrivalled by ELISAs and RIAs. Initially attempts to transfer to MS approaches were hampered due to sensitivity issues which can now be overcome by newer instrumentation, [34,43,44], and a number of successful approaches have now been published (Tables 2 and 3 and Fig. 2). Only a few of these methods include the bioactive metabolites. The critical and defining factors underpinning improvements in speed, sensitivity and reliability are tabulated and discussed below.

**Table 2:** Estrogen (unconjugated) quantification by GC–MS(/MS).

Analyte	Matrix	Extraction Type	V (mL)	Derivatization Agent	Inj V (μL)	Column	MS	Mode (+/-)	LOQ (pg/mL)	Ref
**E1, E2**	Serum	LLE	1	TMSI	1	TR-50MS 50% phenyl polysilphenylene-siloxane 30 m × 0.25 mm (0.25 μm)	Ion Trap EI-MS/MS	+	13-21	[45]
**E1, E2**	Serum	LLE & SPE	0.25	PFBHA PFBO	NS	DB-17HT, 50% phenylmethyl polysiloxane 15 m × 0.25 mm (0.15 μm)	Triple Quad CI-MS/MS	–	0.5	[46]
**E2**	Serum	SPE	1	PFBO PFBHA MSTFA	NS	DB-17 fused silica Dimensions NS	Triple Quad CI-MS/MS	–	1.9	[37]
**E2**	Plasma	SPE	1	PFBC MSTFA	1	50% phenyl-methylpolysiloxane phase 15 m × 0.25 mm (0.25 μm)	Triple Quad CI-MS/MS	–	2.5	[47]
**E1, E2, 16OHE, 16Epi**OHE2,** 16KetoOHE2, 17EpiOHE2, 2,4OHE, 2OHE-3ME, 2,4MeOE**	Urine	SPE	2	EOC PFP	2	MXT-1, Silcosteel-treated stainless steel 30 m × 0.25 mm (0.25 μm)	Single Quad EI-MS	+	20-500	[48]

Chemical Ionization (CI); estrone (E1); estradiol (E2); ethoxycarbonlyation (EOC); Gas Chromatography (GC); 2, 4, 16-hydroxyestradiol (2, 4, 16-OHE2); 2, 4, 16-hydroxyestrone (2, 4, 16-OHE1); 2-hydroxyestrone-3-methyl ether (2OHE-3ME); 16β-Hydroxy-17β-estradiol (16epiOHE2); 16-oxo-17β-estradiol (16ketoOHE2); 16α-hydroxy-17α-estradiol (17epiOHE2); 17α-estradiol (17epiestradiol); Liquid Liquid Extraction (LLE); methoxyestrogens (MeOE); *N*-methyl pyridinium-3-sulfonyl *N*-methyl-*N*-(trimethylsilyl)trifluoroacetamide (MSTFA); Not stated (NS); pentadecafluorooctanoyl chloride (PDFO); pentaflurobenzoyl hydroxylamine hydrochloride (PFBHA); perfluorobenzoyl chloride (PFBO); Solid phase extraction (SPE); Tandem mass spectrometry (MS/MS); *N-(*trimethylsilyl)imidazole (TMSI).

**Table 3:** Estrogen (unconjugated^*^) quantification by LC–MS(/MS).

Analyte	Matrix	Extraction Type	V (mL)	Agent	Inj V (μL)	LC	Column	Mobile Phase	MS	Mode (+/-)	LOQ (pg/mL)	Ref
**E2**	Serum (Pooled)	LLE	0.15	None	20	HPLC	Poroshell 120 SB-C18 2.1 × 50 mm (2.7 μm)	MeOH/H_2_O (+ 0.1% FA or 2.5 mM NH_4_OH)	API 5000 Triple Quad ESI vs APCI vs APPI-MS/MS	+/-	0.14 - 0.68	[49]
**E2**	Serum	LLE	0.2	DMIS	25	UHPLC	phenyl-hexyl 100 x 2.1 mm (1.7 μm)	H_2_O/MeOH+ C_7_H_8_	API 5000 Triple Quad APPI-MS/MS	+	0.5	[50]
**E1, E2** **16OHE2**	Serum (Mouse)	Online LLE	0.1	None	1000	HPLC	Supelcosil LC-8-DB 250 x 4.6 mm (5 μm)	MeOH/H_2_O + C_7_H_8_	API 5000 Triple Quad APPI-MS/MS	-	3 - 5	[51]
**E1, E2**	Serum	LLE	2	PS	20	HPLC	phenyl-hexyl 150 x 2.0 mm (3 μm)	H_2_O: CH_3_CN/H_2_O + 0.1% FA	API 4000 Triple Quad ESI-MS/MS	+	10	[52]
**E1, E2**	Serum	LLE	0.2	DS	50	2D-HPLC	C1 cartridge + Gemini phenyl 100 x 2.0 mm (3 μm)	H_2_O/MeOH + 10 nmol/L H_2_O/CH_3_CN + 10 nmol/L	API 4000 Triple Quad ESI-MS/MS	-	1	[53]
**E1, E2**	Plasma	LLE	0.5	DS	15	HPLC	Synergi, 150 x 2.0 mm (4μMax-RP)	CH_3_CN/H_2_O + 0.1% FA	API 3000 Triple Quad APCI-MS/MS	+	6.3 - 11.9	[54]
**E1, E2** **16OHE** **2,4OHE**	Serum	* LLE	0.5	N/A	10	HPLC	Zorbax C18 250 x 4.6 mm (5 μm)	CH_3_CN/H_2_O	API 3000 Tandem Quad axle ESI-MS/MS	-	10-15	[55]
**E1, E2**	Serum	LLE + Strata X-SPE	1	PED PPZ MPED MPPZ	3	HPLC	YMC-C8 Pro C18 RS 150 x 2.0 mm (5 μm) + 150 x 2.0 (5 μm)	CHCl_3_ /MeOH	API 2000 Triple Quad ESI-MS/MS	+/-	0.55 - 9.2	[56]
**E1, E2**	Serum	LLE	0.5	DS	30	UHPLC	Poroshell 120 SB-C18, 30 x 2.1 mm (2.7 mm) + Zorbax SB-C18, 50 x 2.1 mm (1.8 μm)	H_2_O/MeOH + 0.2% FA	6500 Triple Quad ESI-MS/MS	+	1 - 4	[57]
**E2**	Serum	LLE	0.29	None	5	Micro LC	YMC Triart 50 x 0.5 mm (3 μm)	H_2_O/MeOH + 0.05% NH_4_OH	6500 Triple Quad ESI-MS/MS	-	3	[58]
**E1, E2**	Serum	LLE	0.5	None	100	HPLC	Supelguard LC-8-DB, 20 x 3.0 mm + LC-8-DB, 3.3cm × 2.1 mm (3 μm)	H_2_O/MeOH: CH_3_CN + NH_4_F	5500 Triple Quad ESI-MS/MS	-	0.2 – 0.4	[59]
**E1, E2** ******16OHE2******	Serum	LLE	0.1	DS	20	UHPLC	RP-18 50 x 2.1 mm (1.7 μm)	MeOH/H_2_O + 0.2% FA	4500 Triple Quad ESI-MS	+	5	[60]
**E2** **16OHE2** **MeOE2** **2,4OHE2**	Serum	*LLE	0.1	NMPS	1	nano AQUITY UHPLC	BEH-130 C18 150 x 100 mm (1.7 μm)	H_2_O/CH_3_CN +0.1% FA	TSQ Vantage Triple Quad	+	0.5 - 5	[61]
**E1, E2**	Serum/ urine	LLE	0.01	NBCOCL DNBF	10	HPLC	YMC-Pack Pro C18 RS 150 x 4.6 mm (5 μm)	MeOH /H_2_O	ThermoQuest Finnigan LCQ APCI-MS	-	2000 - 3000	[62]
**E1, E2, 16OHE, 16EpiOHE2, 16KetoOHE2, 17EpiOHE2, 2, 4OHE** **2,4MeOE**	Serum	LLE	0.1	MIS DS PS P	25	HPLC	Ascentis Express C18 150 x 3.0 mm (2.7 μm)	H_2_O/CH_3_CN +0.1% FA	Orbitrap ESI-MS/MS	+	0.2 - 100	[63]
**E1, E2, 16OHE, 16EpiOHE2, 16KetoOHE2, 17EpiOHE2, 2,4OHE** **2OHE-3ME** **2,4MeOE**	Serum (pooled)	LLE	2	None vs C1-NA-NHS	5	UHPLC	XDB-C18 50 x 2.1 mm (1.8 μm)	CH_3_CN A:5%, B:95% +10 mmol/L NH_4_CH_3_CO_2_	TOF APCI vs ESI MS/MS	+ or -	360 - 2340	[64]
**E1, E2 16OHE 2,4OHE2** **MeOE2** **4OHE1 2MeOE1**	Plasma	SPE	0.25	BMP	10	HPLC	Zorbax Extend C18 150 x 4.6 mm (5 μm)	H_2_O/CH_3_CN + 0.1% FA	6420A Triple Quad ESI-MS/MS	+	0.3 – 3.6	[65]
**E2**	Saliva	PPE + Online SPE	0.1	None	200	HPLC	Shim-pack XR-ODS 75 x 3 mm (2.2 μm)	H_2_O/MeOH + 2 mM NH_4_CH_3_CO_2_	API 5000 Triple Quad APCI-MS/MS	+	1	[66]
**E1, E2**	Serum	SPE	0.5 -1	P	100	HPLC	CD-C18 150 x 3 mm (3 μm)	CH_3_CN: CH_3_OH + HCOOH	API 5000 Triple Quad ESI-MS/MS	+	0.5 - 1	[67]
**E2**	Serum	SPE	3	DS	25	HPLC	Zorbax Eclipse ZDB-C18 150 x 2.1 mm (5 μm)	H_2_O/CH_3_CN + 1 mL/L CH_3_COOH	API 4000 Triple Quad ESI-MS/MS	+	1	[68]
**E1, E2** **16OHE2**	Serum	Online SPE	0.1	FMP	300 trap elute	HPLC	Kinetex1 XB-C18 100 x 2.1 mm (2.6 μm)	H_2_O + 2.5% FA /MeOH + 20 mM NH_4_HCO_2_	8050 Triple Quad ESI-MS/MS	+	3 - 7	[69]
**E1, E2, 17epiestradiol, 16OHE, MeOE**	Plasma	SPE	0.5	MPPZ	30	UHPLC	ACE Excel C18-PFP 150 x 2.1 mm (2 μm)	H_2_O/CH_3_CN + 0.1% FA	6500+ Triple Quad ESI –MS/MS	+	2 - 10	[70]
**E1, E2**	Plasma	SPE	0.5 - 2	FMP	20	UHPLC	BEH C18 50 x 2.1 mm (1.7 μm)	Isocratic H_2_O/MeOH + 0.1% FA	5500 Triple Quad ESI-MS/MS	+	2	[71]
**E1, E2,** **16OHE2**	Saliva	SPE	0.25	None	30	UFLC-XR	BEH C18-XP 100 x 2.1 mm (2.5 μm)	H_2_O/CH_3_CN + 0.1 mM NH_4_F	5500 Triple Quad ESI-MS/MS	-	1	[72]
**E2**	Plasma	SPE	0.5	None	NS	UHPLC	HSS T3 C18 100 x 2.1 mm (1.8 μm)	CH_3_CN/H_2_O	TQ-S ESI-MS/MS	+	2	[73]
**E2**	Serum	Online SPE	0.25	None	20	UHPLC	C18 SB 30 x 2.1 mm (1.8 μm)	H_2_O/MeOH	TQ-S ESI-MS/MS	-	3	[74]
**E1, E2** **16OHE2**	Serum	SLE	0.1	None	90	HPLC	Kinetex C18 100 x 3.0 mm (2.6 μm)	H_2_O/MeOH 10% + NH_4_OH (Post Column)	5500 Triple Quad ESI-MS/MS	-	1 - 30	[75]
**E1, E2** **16OHE**	Serum	Deproteination	0.2	None	600	HPLC	LC-8-DB, 3.3 cm × 3.0 mm (3 μm)	MeOH/H_2_O	API 5000 Triple Quad ESI-MS/MS	-	1 - 2	[76]

^*^Data reported for unconjugated estrogen quantification, Atmospheric pressure chemical ionization (APCI); atmospheric pressure photoionization (APPI); 3-bromomethyl-propyphenazone (BMP); 1,2-dimethylimidazole-5-sulfonyl chloride (DMIS); 2,4-dinitrofluorobenzene 2,4-dinitrofluorobenzene (DNBF); dansyl chloride (DS); electrospray ionization (ESI); Estrone (E1); Estradiol (E2); 2, 4, 16-hydroxetradiol (2, 4, 16-OHE2); 2, 4, 16-hydroxetrone (2, 4, 16-OHE1); 2-hydroxyestrone-3-methyl ether (2OHE-3ME); 16β-hydroxy-17β-estradiol (16epiOHE2); 16-oxo-17β-estradiol (16ketoOHE2); 16α-hydroxy-17α-estradiol (17epiOHE2); 17α-estradiol (17epiestradiol); 1-methylimidazole-2-sulfonyl (MIS); Liquid Chromatography (LC); Liquid Liquid Extraction (LLE); methoxyestrogens (MeOE); methanol (MeOH); 1-(2,4-dinitro-5-fluorophenyl)-4,4- dimethylpiperazinium iodide (MPPZ); Mass Spectrometry (MS); 4-nitrobenzoyl chloride (NBCOCL); *N*-methyl-nicotinic acid *N*-hydroxysuccinimide ester (C1-NA-NHS); Not Stated (NS); pyridine-3-sulfonyl chloride (PS); picolinoyl carboxylate (P); *N*’-(5-fluoro-2,4-dinitrophenyl)-*N*,*N*-dimethyl-1,2- ethanediamine (PED); 1-(2,4-dinitro-5-fluorophenyl)-4-methylpiperazine (PPZ); Solid phase extraction (SPE); trifluoracetic acid (TFA); tandem quadrupole mass spectrometry (TQ-S) 1,2- dimethylimidazole-5-sulfonyl chloride; 2-fluoro-1-methyl- pyridinum *p*-toluene sulfonate (FMP); Ultra Flow – LC (UF-LC); Ultra high performance–LC (UHPLC).

### 2.3 Methods of detection and quantitation

Mass spectrometers interfaced with GC and LC have both been successfully applied to analyse estrogens in plasma and/or serum, with a number of validated methods reported, Table 2, following the general principles in Fig. 2. It is important to recognize that when studying a family of related molecules that several estrogens may fragment to the same ion, and efficient chromatographic separation remains essential to avoid isobaric interferences. This happens for example between isomers and isotopologues, notably [M + 2], being a particular problem with groups of molecules interconverted between ketones and alcohols by oxidation/reduction. These possibilities must be planned for and thus potential sources of interference excluded upon method validation. For MS, single quadrupoles were initially developed allowing one mass filter to be applied for selection of a single *m/z* ratio in an approach known as selected ion monitoring (SIM). Advances in this technology led to the introduction of triple quadrupoles, allowing double mass filtering of initial precursor ions and their breakdown fragments (product ions) – known as multiple reaction mode (MRM). Triple quadrupole MS operated in MRM rather than conventional SIM provide a much higher selectivity, with less interference from co-eluting matrix components and thus increased signal to noise ratio. This has allowed enhanced selectivity over wide dynamic ranges and improved accuracy and precision of assays. Quantitation with high resolution or accurate mass analysers is possible but still in its infancy, held back in some cases by poorer quantitative performance due to narrower dynamic ranges [64]. Quantitative performance of these diverse analysers is variable, with Time-of-Flight instruments to date performing less well (Table 3) but some valuable methods coming forward with Orbitrap® technology [30,63]. Their value in the field for structural identification and elucidation of fragmentation is however extremely important and well established [70,77].

### 2.4 GC–MS (/MS)

In GC, the mobile phase is an inert gas (usually helium) and the stationary phase is a viscous liquid that coats the walls of the capillary column. Analytes must vaporize and then dissolve into the stationary phase upon injection onto the GC column. Subsequently they are volatilized, and efficient phase transfer of steroids usually requires chemical derivatization. The derivatized steroids are resolved on the capillary column based on their relative affinities for the stationary phase and the temperature gradient applied to the GC oven. Stationary phases with phenyl groups have been most commonly used for estrogen analysis, as П- П interactions with the phenolic A-ring enables resolution of more challenging mixtures. Historically and still today greater resolving power is afforded with GC than LC, an important factor for improving isomer resolution and accurate and specific quantification.

Chemical derivatization is applied with a range of reagents reported in Table 2. For MS analysis, both electron impact (EI) and chemical ionization (CI) have been used and of these, CI in conjunction with tandem MS is favoured in the literature. This is due to improved sensitivity brought about by electron capturing halogenated derivatization reagents. Accordingly the vast majority of GC–MS/MS approaches capable of detection of estrogens at low concentrations employ negative ion mode, although positive ionization has been employed in conjunction with ion-trap technology successfully achieving a lower limit of quantitation (LOQ) of 13–21 pg/mL [45]. However, this would not be sufficiently low for certain patient cohorts, Table 1. Urinary analysis of estrogens and their bioactive metabolites by GC typically involves extended sample preparation (two-step extraction) [48]. For analysis of estrone and estradiol in serum, these types of extractions in conjunction with derivatization show reliable detection, however extensive sample preparation can be required, for example with both liquid-liquid extraction (LLE) and solid phase extraction (SPE) for estrone and estradiol, to allow detection at 0.5 pg/mL in 250 μL of rodent serum [46]. Approaches using SPE are most efficient for single step methods, with applications reporting limits of 1.9 pg/mL from 1 mL of serum from post-menopausal women [37] and 2.5 pg/mL in an application note from 1 mL serum [47]. Notably methods have not been reported for catechol and hydroxyl metabolites of estrogens in plasma by GC, although in principle this should be achievable as they have been detected in urinary samples [48]. Although GC–MS/MS inherently allows enhanced chromatographic resolution vs LC-MS(/MS), its routine application suffers from time consuming runs (30 min – 1 h), extensive sample preparation, use of high temperatures that may be detrimental to thermo-labile compounds and complex fragmentation of precursor ions within the MS source. LOQs for a subset GC methods remain marginally outside the clinical range, with only one study in range reaching an LOQ of 0.5 pg/mL. This approach has still to be tested in human serum as opposed to rodent and relies on extensive sample preparation [46]. LC–MS(/MS), using softer ionization techniques, are more likely to generate charged molecular ions. Thus, there has been much interest in bringing LC–MS(/MS) methods to the fore. This may provide higher throughput, although it is worth debating whether adequate sensitivity can be achieved without derivatization.

### 2.5 LC–MS (/MS)

LC–MS/MS is fast becoming the favoured approach for steroid analysis in clinical laboratories worldwide consequent to technological advances in ion formation, transfer and detection. For the applications discussed here, reversed phase chromatography is almost exclusively used, using LC columns with hydrophobic stationary phase in conjunction with a polar mobile phase. C18 columns [49,[55], [56], [57],^61^,^63^,^64^,^69^,^70^,^72^,^78^] with their enhanced retention capabilities and robust, consistent manufacturing quality are preferred. Chemical alterations to bonded stationary phases, again exploiting interactions with the aromatic ring, can improve distinction of isomeric structures, typically required for the estrogen metabolites. Efficiency of separation can be enhanced by use of smaller particle sizes and longer columns, parameters which are inversely proportional to chromatographic efficiency [70]. Pairings of a wide variety of mobile and stationary phases are cited each with their own individual benefit. The careful choice of gradient elution parameters improves robustness of the assay, incorporating time for equilibration, elution and column cleaning. While ballistic gradients are attractive in reducing analysis times and in the cleaning phases, more subtle gradients are often necessary to resolve isomers. Normally combinations of either methanol or acetonitrile and water are used [49,54,70] with only one report using both acetonitrile and methanol [56].

Given the low abundance of estrogens, it is unsurprising to see the use of triple quadrupole instruments, with their improved signal to noise, dominating over that of single quadrupole systems (Table 3). Positive-mode electrospray ionization (ESI) analyses are most prominent, at least for derivatized samples, Table 3, but alternative soft ionization modes are also reported, namely atmospheric pressure chemical ionization (APCI), and the more recent atmospheric pressure photoionization (APPI) [49,79]. Estrogen analyses are reported in both positive and negative ionization modes dependent on the charge due to (de)protonation or coupled by derivatization. Mobile phase modifiers such as formic acid, ammonium formate or acetic acid are frequently added.

Limits of quantitation by LC–MS/MS are reported over a wide range of 0.14–3000 pg/mL for estrone and estradiol. Inclusion of metabolites to create assay panels might be associated with a reduction in sensitivity due to lower dwell times for each scan but this is not that apparent in the applications reported in Table 3. Generally, 0.1–2 mL of serum or plasma are required although 0.5 mL or below is desired for routine collection without excessive blood loss. The limits and volumes differ somewhat between ionization methods, Table 3. Examples include low limits of detection (0.5–2.4 pg/mL) for metabolite panels in a study of breast cancer patients [65], and again in another study of pulmonary hypertensive patients (2–10 pg/mL) [70]. Negative mode is more commonly used in non-derivatized samples [36,51], capitalizing on the presence of a phenol within the estrogen structure with methods generally reporting limits of ∼1 pg/mL for estrone and estradiol. However ionization of non-derivatized estrogens in ESI mode occurs within charged droplets in competition with alternative species present in endogenous mixtures, which can cause ion suppression and this parameter must be evaluated in method development [80]. As an alternative, APCI is less liable to ion suppression as is APPI, the latter using photons from a discharge lamp to aid ionization of molecules. Reported applications of APCI methodologies for estradiol analysis have low limits of 0.5 pg/mL in serum [49] and 1 pg/mL in saliva [66] with limits for APPI reported as 3–5 pg/mL for estrone, estradiol and estriol in mouse serum [51]. Rahkonen et al presented a comprehensive comparison of all ionization modes and polarity combinations for estradiol analysis clearly demonstrating APPI in negative mode to have the lowest LOQ at 0.14 pg/mL. Using this approach, low concentrations in pooled serum were detected using ammonium hydroxide as an additive, Table 3 [49]. Upon application to a clinical cohort (200 μL), a DMIS derivatization approach in conjunction with APPI was applied by the same group reporting limits in serum of 0.5 pg/mL [50]. Notably addition of 2D chromatography boosts sensitivity for estrone and estradiol reducing LOQs tenfold [53] and integration of this with APPI might allow a further boost in quantification capabilities. As of yet APPI is not widely reported or available and, although advantages of APCI/APPI over ESI exist, the majority of routine analytical assays applied in clinical laboratories use ESI, creating a deterrent to regular exchange between MS interfaces and hindering their extended application.

Alternative LC approaches such as ultra-flow LC (UFLC-MS) might be applied allowing reduction of flow rates (1–100 μL/min) combined with columns of smaller dimensions (ID; 0.1 and 1.0 mm). Advantages suggested include the ability to use smaller volumes of solvents which is of economic and environmental benefit, wider dynamic ranges and improved sensitivity [81]. A further application of microflow was introduced as the Ion Key® source from Waters, allowing direct infusion of microflow-LC into the MS with reported advantages of improved sensitivity, chromatographic performance and importantly robustness. This has been exemplified in a technical report for estrone, estradiol and estriol, with LOQs for non-derivatized steroids of 1 pg/mL, using negative-ESI with ammonium hydroxide as a mobile phase modifier for deprotonation. The authors here suggest clear analytical advantages over non-derivatized methods by conventional ESI-LC–MS/MS methods [81] but this is not always the case as shown in Table 3. In another study by Wang et al using nano-LC technology for analysis of serum in men, LOQs reached 0.5 pg/mL for estradiol and its metabolites except in the case of catechol estrogens whose limits were 5 pg/mL [61]. However, again this approach is not yet in routine use. Overall, a wider application of LC–MS/MS than GC–MS/MS for quantification of estrogen and its bioactive metabolites in plasma, serum and more recently in saliva has been demonstrated.

### 2.6 Future perspectives on analytical technology

Simultaneous analysis of estrogen metabolite panels in biological matrices constitutes a difficult task. This subgroup of steroids, with their low abundance, has created and still presents challenges for analysts in the search for high-throughput, facile and sensitive assays. With the advances in technology, this is now within grasp, but to quantify these molecules the sample volumes required are still relatively large and considerably more than for other steroids such as androgens. In the future, aside from advances in standard GC or LC triple quadrupole technology, coupling of MS/MS to advanced chromatographic technologies such as supercritical fluid chromatography, ion mobility MS and micro-LC/nanospray might permit improved sensitivity and shorter run times. Proponents of supercritical fluid chromatography suggest that this separation method harnesses the advantages of both LC and GC, commonly employing methanol/carbon dioxide linear gradients as a mobile phase and resulting in faster separations and higher efficiencies than conventional GC. This non-polar solvent system has a low backpressure, allowing higher flow rates than LC due to the viscosity of the mobile phase being more similar to gas rather than liquid. Application to a panel of 15 estrogen metabolites in urine and serum was trialled for estrone, estradiol, estriol, 16-hydroxy, 16-ketohydroxy, 2-hydroxy, 4-hydroxy, 2-methoxy and 4-methoxy–estrogens showing fast run times and 5 pg quantification limits [82]. This has yet to be validated for routine use and the availability of instrumentation is still restricted. Ion mobility mass spectrometry (IMS) provides an interface between the LC and MS/MS systems allowing separation of ions in the gas phase. Discrimination is based on their mobility differences in either high vs low electric fields and is dependent on their collisional cross sections. There are three main forms of IMS, drift tube (DT-IMS), travelling wave (TW-IMS) and field asymmetric/differential (FA/D-IMS). Of these, DIMS has been applied successfully to tissue samples for separation of estrone, estradiol and estriol from American eels [83]. In this case, ion mobility allowed efficient separation of structural isomers whilst reducing background noise over conventional ionization methods. For quantitation, IMS in principle may allow better signal to noise within the detector since species creating contemporary noise maybe separated from analytes through differential mobility. Isomeric estrogens have extremely similar mobility but the potential exists for derivatization to exaggerate structural difference and subsequently increasing separation between structural isomers [84]. As of yet this approach has not been commonly employed, perhaps due to a lack of dedicated systems in academic laboratories.

### 2.7 Sample preparation

Prior to quantification estrogens must be efficiently extracted from the matrix of choice. In the case of LC–MS/MS analysis, ion suppression arises due to sample components, such as phospholipid and salt interference. Phospholipids remain the number one cause of diminished signal responses for analytical applications by LC–MS/MS. GC, however, does not suffer from this phenomenon due to the high energy nature of its ionization source, although remaining matrix components can cause undesirable deposits in the injector, start of the column or in the source and will lead reduced sensitivity and poor peak shapes. For either approach, sufficient removal of interfering compounds and lowering of background noise by sample pre-treatment is of paramount importance when approaching estrogen assay development.

#### 2.7.1 Liquid-liquid extraction (LLE)

LLE provides an inexpensive approach to extract estrogens from the sample matrix exploiting their relative solubility in organic solvents. LLE has been used extensively and as a result is most common for the analysis of estrone and estradiol in serum [35,59]. Ultimate recovery and suppression of LLE approaches are dependent on the choice of extraction solvent. Solvents for extraction of estrogens into the organic phase include methyl *tert*-butyl ether (MTBE), ethyl acetate, diethyl ether, dichloromethane or mixtures of these organic solvents. Ethyl acetate is most commonly reported affording high recoveries [85,86] and alongside MTBE, it yields a clean extract that avoids precipitation upon derivatization [87,88]. From Table 3, it can be seen LLE has been extensively applied to estrone and estradiol assays reporting low detection limits (0.14–5 pg/mL). However, for bioactive metabolites results are variable with higher limits than alternative sample preparation techniques (5–360 pg/mL). Additional drawbacks of LLE for routine testing also relate to its manual nature, commonly being more time-consuming and potentially exposing the analyst to high volumes of organic solvents. Sample loss through transfer between test tubes and plastic plates has been noted using LLE possibly contributing to inter-day imprecision [49].

#### 2.7.2 Solid phase extraction (SPE)

Off-line SPE is an attractive alternative to LLE. It is often employed for analysis of estrogens in water (i.e. for processing larger sample volumes), but also with effective application in the clinical setting in saliva, serum and plasma [54,68,72]. SPE extraction cartridges come embedded with a range of solid packing materials, which chemically separate the components of interest from the biological samples. Varieties of bed are commercially available containing reversed, normal, ion exchange or adsorption packing materials. For recovery of estrogens from aqueous sample matrices, reversed and ion exchange phases are recommended and are reported to be effective for clean up of plasma samples, Table 3. In principle, an SPE column containing an alternative packing material to the chromatography column holds advantages in improving sample clean up. SPE columns used for estrogen analysis often have C18 beds, but many commercial materials also exist such as Oasis HLB®; most have hydrophobic characteristics optimal for interactions with the lipophilic features of steroid hormones. HLB® operates over a wide range of pH values suitable for many compound classes. Choice of SPE column is based on achieving high recovery with low ion suppression, which can be difficult to achieve with complex matrices such as plasma [71]. A study by Faqehi et al 2016 suggested the use of Oasis MCX®, a cartridge housing a mixed mode cation exchange reverse phase bed, provides opportunities for additional sample clean up prior to the elution of the estrogen and this has been shown effective for a panel of the bioactive estrogens, including metabolites upon optimization of wash steps [70]. Other groups suggest the use of C8 polypropylene columns conditioned and cleaned with 0.1% TFA improved recovery and diminished ion suppression for a panel of 10 estrogens [65]. Moving forward with SPE, newer products eliminate the need for conditioning and equilibration steps and availability of 96-well plates allow potential automation for robotic liquid handling systems. The main disadvantage with SPE for routine clinical analyses associates with the cost, as cartridges remain expensive. Moreover, coupling SPE and derivatization can introduce undesirable transfer steps and also losses depending on the type of collection container required to avoid adhesion (glass vs plastic). Glass inserts for 96-well plates are expensive and only available for lower elution volumes. On-line SPE methods are available although less frequently reported as they can be complicated to develop without compromising the analytical chromatographic step [89]. However once the elution programme is optimized, directly linking the extraction processes to LC–MS/MS can improve recovery and sensitivity and minimize manual sample manipulation [90].

#### 2.7.3 Supported liquid extraction (SLE)

Supported liquid extraction (SLE) opens doors to new approaches for extraction but as yet methods for estrogen analysis have been scarcely published, unlike with other steroids [91]. This strategy shows promise in company application notes [92] with successful application to androgen profiling for diseases such as congenital adrenal hyperplasia [93]. SLE applies the same solvent affinity principles as LLE whereby analytes are separated based on their partitioning into one solvent over another immiscible solvent and employs similar solvents. The support material consists of diatomaceous earth, a natural silica product (∼90% silica), being an ideal material to absorb aqueous samples. This technique allows shorter load, wait and elute protocols to the generic SPE approaches, and the conditioning and equilibration steps of the cartridge bed are not needed. However, options for sample clean-up are limited in comparison to SPE. Again, SLE can be fully automated in 96-well formats but again there are challenges in interfacing with containers suitable for derivatization. One application for analysis of estrone, estradiol or estriol from 100 μL of plasma in the SLE 96 well format shows potential with low limits of 1, 3 pg/mL for estrone, estradiol respectively and 30 pg/mL for estriol. This extraction method should now be tested with the wider panel of metabolites on more sensitive MS platforms.

#### 2.7.4 Derivatization

Derivatization can be necessary prior to analysis of estrogens by MS, but with different goals for GC and LC. In the case of GC it is necessary to enhance volatility often with the introduction of halogen atoms, also enhancing sensitivity of CI approaches [79]. For LC, derivatization is often employed to aid formation of charged ions or generate permanently charged species. This increases sensitivity, and the greater mass of the molecular ion holds further benefits for specificity. In GC–MS/MS the process sometimes adds poorly volatile reagents which cannot be easily removed. In both GC and LC, derivatization reagents can build up in the chromatographic column or within components of the mass spectrometer, thus decreasing assay robustness. In LC, this may be addressed by diverting the initial flow prior to analyte elution to waste, removing polar reagents and maintaining a clean interface and source within the mass spectrometer. In GC frequent cleaning of the inlet liner will be required.

##### 2.7.4.1 GC approaches

In GC–MS/MS derivatization at the 3′ position of the A-ring is favoured as reactions at the saturated aliphatic D ring largely do not improve sensitivity over non-derivatized samples illustrated by pentafluoropropionyl (PFP) or trimethylsilyl (TMS) derivatives for water analysis by GC–MS/MS [94,95]. The generation of PFB derivatives is the most commonly reported approach for estrone and estradiol analysis in serum, but cumbersome sample preparation steps have, however, hampered routine use [37,46,96].

##### 2.7.4.2 LC approaches without derivatization

Development of analytical workflows of sufficient sensitivity without derivatization remains challenging for clinical applications of estrogen analysis, although they are desirable with sample preparation being shorter with a lower chance of introducing manual varation. Moreover, automation of derivatization by commercial robots is challenging to couple with robotic SPE/SLE workflows. However, a number of methods for underivatized estrone and estradiol using LC–MS/MS are beginning to surface, (Table 2) as instrument technology improves. Methods achieving LOQs comparable with derivatization approaches have been reported using ammonium fluoride or ammonium hydroxide as mobile phase modifiers, promoting the formation of negative ions [59,72]. Recent analyses of estradiol report low LOQs, for example of 2 pg/mL using an UHPLC System coupled to a Xevo TQ-S [73]. Methods without derivatization are yet to be extended to include bioactive estrogen metabolites. If optimized successfully, validation of such assays would permit simplified sample preparation with the possibility of higher precision and throughput.

##### 2.7.4.3 LC approaches with derivatization

Derivatization remains necessary for the majority of LC–MS assays of estrogens, overcoming poor ionization, limiting ion suppression and boosting signal intensity at low abundance. In reactions reported, introduction of easily ionizable groups or pre-charged moieties improves sensitivity and permits the use of lower volumes of sample. As in GC–MS, the hydroxyl group of the phenolic A ring in the 3′ position is usually targeted for the entire analyte panel. Successful derivatization methods commonly reported for analysis of estrone and estradiol include use of dansyl chloride [68,92,97,98], *N*-methyl-nicotinic acid *N*-hydroxysuccinimide ester [64], 2-fluoro-1-methylpyridinium-*p*-toluene sulfonate [71], methyl-1-(5-fluoro-2, 4-dinitrophenyl)-4,4-dimethylpiperazine [56,70], isomers of 1,2-dimethylimidazole-sulfonyl chloride [63,99], picolinoyl carboxylate [67], pyridine carboxylates [100], pyridine-3-sulfonyl chloride [52] and *p*-nitrobenzyl chloride [101]. From these, dansyl chloride has been the most common approach. However, the specificity of the fragment ions of dansyl chloride derivatives is hindered for isobaric estrogen metabolite species since the product ions generated are identical, hailing from the derivative [63,97,98]. This is similar for alternative derivatives such as BMP [65], whereby methyl-propyphenazone derivatives generate identical product ions for seven estrogens whilst differing by *m/z* 15 for the catechol metabolites. This source of non-specificity has been partially overcome by use of MPPZ and C1-NA-NHS, yielding a range of product ions, but they remain identical for certain groups of metabolites [64,70], since isomers undergo similar fragmentation patterns. Therefore, thorough evaluation of chromatographic methods becomes a necessity, to eliminate possible co-elutants that may be mis-identified leading to reporting of false positives. It should not be forgotten that estradiol and estrone only differ by 2 mass units so ^13^C_2_ isotopologues used as internal standards will cross-signal if product ions are identical. It is not uncommon for multiple aliphatic and phenolic hydroxyl groups to be derivatized within the reaction especially within 16-hydroxy- and catechol estrogens [56,102], yielding either doubly or triply charged species or isomeric derivatives. Finally, if derivatization is deemed necessary the stability of derivatives should be considered and must be studied to ensure practical laboratory workflows e.g. FMP derivatives degrade following 48 h at −20 °C but remain stable within −80 °C storage [71]. MPPZ derivatives show minimal degradation (<15%) upon storage for 8 days in the autosampler and for up to 31 days in −20 °C storage [70]. Dansyl chloride derivatives have also been reported to be stable over a 7 day period in patient plasma [54]. However, in the majority of current literature, this information is lacking for derivatization approaches. Derivatization techniques are still less preferred in the clinical setting, due to the addition of another complexity within sample preparation inevitability contributing toward data variability and increased turn-around time.

#### 2.7.5 Internal standards

An important feature of MS analytical methods for estrogen quantification is the availability of stable isotope labelled internal standards (IS) giving a retention time match to both derivatized and non-derivatized estrogens. Addition at constant concentrations within the assay accounts for extraction loss at all stages. ^13^C-labels allow additional selectivity over deuterium-labelled standards, since they are highly unlikely to be removed during processing. Deuterium can be removed through either deuterium-hydrogen exchange under acidic conditions or, depending on the positions of the labels, during derivatization reactions. By GC and LC, the retention time of ^13^C-labelled standards are well aligned whereas deuterated IS may differ slightly, probably due to isotope effects on hydrogen *vs* deuterium bonding interactions with the stationary phase. The slight differences in retention time that arise with deuterium labels are exaggerated when the number of heavy labels is increased potentially leading to less accurate quantitation with less specific interpretation of matrix effect [103]. However deuterium labelled standards are applied in a number of studies generally being less expensive in comparison to the ^13^C labelled versions [51,53,57,63,65]. Retention of the stable isotope labels in the product ion is desirable to enhance specificity, but labels can be lost in fragmentation, leaving product ions identical in *m*/*z* to the analyte. C_3-6_ labelled standards are now available for all estrogens shown in Fig. 1. Multi-labelled standards, preferably in excess of two labels, e.g. ^13^C_3_ and ^13^C_6._ should be utilized, to avoid interference with natural isotopologues [61,70].

## 3 Conclusion

As this review highlights, there is no universal method for estrogen analysis, however the wide range of approaches developed over the past 10–15 years allows us to nudge closer to the possibility of routine investigation and monitoring of estrogen sensitive diseases. On comparison of technologies available, methods by GC–MS initially came to the field and currently offer a range of LOQs between 0.5–21 pg/mL for estrone and estradiol in serum, plasma and urine, with 20–500 pg/mL for their metabolites. However, despite efficient resolution of isomers, GC–MS(/MS) is less favoured requiring more extensive sample preparation and the absolute requirement for derivatization limiting automation. Developments in LC–MS/MS arose more recently, with technology still advancing, offering the possibility of lower detection limits of 0.14 pg/mL for standalone estradiol analysis with a range more commonly between 0.5–21 pg/mL for estrogens in panel assays. Although UHPLC may reduce analysis times in conjunction with MS, applications show similar limits of detection to conventional HPLC and GC. SPE and SLE extraction methods will likely lead the way forward in clinical assays due to the possibility of automation. Applications involving derivatization are not universally superior with a number of methods not requiring derivatization now emerging that display similar or even lower detection capabilities. Therefore, development of approaches without this step should be considered on newer triple quadrupole instrumentation. In this setting APCI and APPI modes have yet to be explored for the full metabolite panels. Irrespective of analytical technology used, the importance chromatographic development must not be understated due to estrogenic isomers, stereoisomers and isobaric confounders. Combinations of on-line SPE, high-resolution LC and MS approaches may shape the future for automated approaches; ion mobility might also provide a key approach for separation of isomers, enhancing structural confirmation in cases where shared product ions arise.

In conclusion, advancing research into health and disease in clinical cohorts extending to children, men, and pre/post-menopausal women for disease diagnostics and monitoring means limits of analytical methods are constantly being tested. MS has established its place at the forefront of research for estrogen quantification in clinical laboratories, with LC–MS/MS beginning to show potential for routine applications.
